# Accounting for Age Uncertainty in Growth Modeling, the Case Study of Yellowfin Tuna (*Thunnus albacares*) of the Indian Ocean

**DOI:** 10.1371/journal.pone.0060886

**Published:** 2013-04-23

**Authors:** Emmanuelle Dortel, Félix Massiot-Granier, Etienne Rivot, Julien Million, Jean-Pierre Hallier, Eric Morize, Jean-Marie Munaron, Nicolas Bousquet, Emmanuel Chassot

**Affiliations:** 1 Unité Mixte de Recherche 212 Ecosystèmes Marins Exploités, Institut de Recherche pour le Développement, Sète, France; 2 Unité Mixte de Recherche 985 Ecologie et Santé des Ecosystème, Université Européenne de Bretagne, Rennes, France; 3 Indian Ocean Tuna Commission, Victoria, Seychelles; 4 Unité Mixte de Recherche 6539 Laboratoire des Sciences de l′Environnement Marin, Institut de Recherche pour le Développement, Plouzané, France; 5 Department of Industrial Risk Management, Electricité De France, Chatou, France; Aristotle University of Thessaloniki, Greece

## Abstract

Age estimates, typically determined by counting periodic growth increments in calcified structures of vertebrates, are the basis of population dynamics models used for managing exploited or threatened species. In fisheries research, the use of otolith growth rings as an indicator of fish age has increased considerably in recent decades. However, otolith readings include various sources of uncertainty. Current ageing methods, which converts an average count of rings into age, only provide periodic age estimates in which the range of uncertainty is fully ignored. In this study, we describe a hierarchical model for estimating individual ages from repeated otolith readings. The model was developed within a Bayesian framework to explicitly represent the sources of uncertainty associated with age estimation, to allow for individual variations and to include knowledge on parameters from expertise. The performance of the proposed model was examined through simulations, and then it was coupled to a two-stanza somatic growth model to evaluate the impact of the age estimation method on the age composition of commercial fisheries catches. We illustrate our approach using the saggital otoliths of yellowfin tuna of the Indian Ocean collected through large-scale mark-recapture experiments. The simulation performance suggested that the ageing error model was able to estimate the ageing biases and provide accurate age estimates, regardless of the age of the fish. Coupled with the growth model, this approach appeared suitable for modeling the growth of Indian Ocean yellowfin and is consistent with findings of previous studies. The simulations showed that the choice of the ageing method can strongly affect growth estimates with subsequent implications for age-structured data used as inputs for population models. Finally, our modeling approach revealed particularly useful to reflect uncertainty around age estimates into the process of growth estimation and it can be applied to any study relying on age estimation.

## Introduction

Estimating individual age of living beings is fundamental in ecological studies. For some time, growth, survival and reproductive characteristics have been shown to be age-dependent as ageing generally conducts to decreasing fertility and increasing mortality with advancing age [1]. Age information currently forms the basis of population dynamics models used for the management of natural populations [2]; [3]. For example, quantitative stock assessment models use age information to relate distinct demographic rates to age classes [4]. Although some stock assessment models may link demographic processes to size or stage classes rather than to age, the transition rates between such classes are generally based on estimates of age-dependent growth.

Age estimates are generally based on counts of periodic growth increments deposited in the hard and skeletal tissues of vertebrates that are capable of recording some biological phenomena, e.g. mammalian teeth, tortoise shell, bird feather, insect cuticle, bivalve shell, and coral skeleton [5]; [6]; [7]; [8]; [9]. For bony fishes, different calcified structures can be used for age determination, i.e. scales, vertebrae, otoliths, and spines [10]; [11]. Over the last decades, otoliths have become an invaluable tool for ageing fish. This is because the otoliths of many temperate and tropical fish species exhibit seasonal and daily growth marks and continue to grow even when somatic growth is slowed or naturally stopped [12]; [13]; [14]. Consequently, counts of otolith increments provide a direct estimation of fish age.

However, otolith reading involves some interpretation by the reader which can lead to imprecision and bias in age estimation [15]; [16]. This may subsequently affect estimates of demographic and biological parameters of populations and eventually modify the perception of stock status and the associated management advice [17]. Errors in interpreting and counting daily increments can initially be related to otolith preparation for reading. In particular, some increments may be “lost” at the otolith nucleus, i.e. the core, and edge [18]. Otoliths can also exhibit discontinuities and zones of overlap that result in some increments being omitted or counted more than once. Reading errors are generally higher for older fish because they have more increments for counting and increments tend to get narrower with the distance from the nucleus when fish approach their asymptotic length [19]; [20]. In addition, growth increments may not always have consistent daily deposition, i.e., sub-daily increments and discontinuities in the accretion rate may occur due to stress, reproduction, and environmental conditions, which may result in biased age estimates [21]; [22].

Multiple independent readings of the same otolith have often been used to estimate the consistency and reproducibility of reading method, to compare the readers skill and to assess the imprecision in age estimates [23]; [15]. In addition, validating the frequency of increment formation is crucial to obtain an accurate fish age. Mark-recapture experiments of fish that have been chemically tagged with oxytetracycline (OTC) are considered to be one of the best methods for validating age interpretation [24]; [25]. After injection, the OTC is rapidly incorporated into the otolith and results in a permanent mark at the increment that formed at the time of tagging. This mark is visible under fluorescent light. The number of growth increments formed after the mark can then be compared to the time between tagging and recapture, i.e. time-at-liberty, to test the hypothesis of periodic increment formation.

Yellowfin tuna (*Thunnus albacares*, Bonnaterre 1788) is an epipelagic species that is widely distributed in the tropical and subtropical waters of the major oceans. It has been commercially harvested since the early 1950s [26]. Over the last decades, global catches of yellowfin have steadily increased to more than 1.4 million 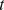 in 2003 with a mean value of 1.2 million 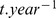 during the 2000s [27]. In the Indian Ocean (IO), the stock of yellowfin is exploited by diverse array of small-scale, semi-industrial, and industrial fisheries and currently represents about 30% of the global catch of yellowfin [28]. The management and conservation of yellowfin in the IO is under the jurisdiction of the Indian Ocean Tuna Commission (IOTC) and relies on the assessment of the stock status through age-structured population dynamics models [29]. Growth of the Indian Ocean yellowfin has been the focus of several studies based on modal progression analysis [30]; [31]; [32]; [33] and direct ageing of scales [34], vertebrae [35], and otoliths [20]. Nevertheless, much uncertainty currently remains on the growth to be considered in yellowfin stock assessment due to the lack of age validation in past studies and to the difficulties associated with tracking yellowfin cohorts over time due to their extended spawning periods (IOTC 2011). In addition, historical studies on yellowfin growth have relied on the classical Von Bertalanffy model (1938), which assumes a constant growth rate over the full lifespan of the fish, while most recent studies support a two-stanza growth curve that is characterized by a significant change in the growth rate between juveniles and adults [36]; [37]; [32].

Since the 1990s, Bayesian modeling approaches have attracted growing interest in the fields of applied ecology and environmental sciences [38]. The Bayesian framework offers the advantage of incorporating expert judgment and supplementary information into the statistical data analysis in a rigorous and consistent manner [39]; [40]. This is particularly appropriate for fisheries science where data are almost always partially observed and include some measurement errors or uncertainties. Bayesian models have been used to make inferences about fish growth [41]and spatio-temporal population dynamics [42] and to provide scientific advice for fisheries management [43]; [44]; [45]. Hierarchical Bayesian modeling is particularly powerful as it can exploit of a diverse range of information sources and draw inferences on large numbers of latent variables and parameters that describe complex relationships by decomposing the phenomenon in a series of submodels [38]; [46]. Hierarchical models can make powerful inferences because they account for both observation and process errors, the latter being generally attributed to stochastic environmental variations [40].

Using yellowfin as a case study, we describe an approach for estimating the individual ages of fish from repeat readings of otoliths that takes account of associated uncertainties. We apply our model to an original age-length dataset collected through large-scale mark-recapture experiments conducted in the IO between 2005 and 2012. Firstly, an ageing error model was developed to explicitly represent the sources of uncertainty associated with age estimation. Developed in a hierarchical Bayesian framework, expert judgment was included in the model through the choice of stochastic error structure and informative prior density functions. A simulation framework was developed to evaluate the accuracy of the model, its limitations and its relevance to the traditional ageing method that estimates age based on an average increment count. Secondly, the ageing error model was coupled to a two-stanza somatic growth model in order to propagate age uncertainty into growth parameter estimates. The use of Bayesian modeling allowed for the integration prior understanding of growth parameters from expertise and historical observations of length and growth for yellowfin. Finally, we evaluated the impact of the age estimation method on the catch age composition of the commercial fisheries targeting yellowfin in the IO. With this case study, we provide a flexible statistical framework that accounts for age-related uncertainty in growth modeling and addresses an applied ecology problem.

## Materials and Methods

### Data collection

Otoliths and length data were collected throughout the Regional Tuna Tagging Project (RTTP), a large scale mark-recapture program, and the West Sumatra Tuna Tagging Project (WSTTP), a simple capture program, that were carried out by the IOTC. The tagging operations of RTTP were conducted between 2005–2007 on two pole-and-line vessels that were chartered to operate in the Western Indian Ocean. Field operations consisted in catching tunas, tagging them on a vinyl-covered cradle, measuring their fork length (fish length from the front to the fork in the center of the tail; 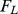) through marks printed directly on the cradle and finally releasing them at sea [47]. The tagging operations of RTTP were conducted between 2005–2007 on two pole-and-line vessels that were chartered to operate in the Western Indian Ocean. Field operations consisted in catching tunas, tagging them on a vinyl-covered cradle, measuring their fork length (fish length from the front to the fork in the center of the tail; 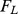) through marks printed directly on the cradle and finally releasing them at sea [47]. The fish were tagged with dart tags inserted into the musculature, below the second dorsal fin. The date and geographic location were recorded for each tag event. Some fish received a OTC injection, an antibiotic that is rapidly incorporated into calcified parts such as bones, scales, and otoliths and leaves a permanent fluorescent mark in the growth increment being formed at the time of tagging. Depending on fish size, between 1.5–3 mL of OTC were injected with a syringe in the intramuscular region of the back [47].

Recovery operations took place across the entire IO basin between 2005–2012. The majority of reported recoveries came from fish caught by the European purse seiners (IOTC 2011). 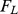 of recovered fish was measured with calipers or a tape measure to the nearest 0.5 cm. The accuracy in the date and location of recaptures is dependent on the place and the process in which the tag is recovered. About 20% of the recoveries were made during purse seine fishing operations, which resulted in the recovered fish being associated with only one position and date. In contrast, tunas recovered during the unloading of purse seiners could be associated with several dates and catch locations due to the process of storing tunas in refrigerated wells that contain up to 5 sets collected over an entire fishing trip. The recovery can also occur downstream of the unloading process or in the canneries. The range of dates associated with each recapture was derived from logbook data and well maps and conducted in close collaboration with the IOTC and the purse seine fishing industry.

The WSTTP program was conducted in August 2007 on a pole-and-line vessel in the off western Indonesia. The fork length of fish measured either in a marked cradle or using a calipers, and the precisely date and geographic location were recorded for each fish caught.

### Otolith sampling, preparation and reading

Sagittal otoliths were collected from 128 yellowfin recovered in the Western Indian Ocean through the RTTP, including 124 OTC-tagged fish, and measuring between 43 to 85 cm 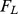 at tagging and 47.9 to 146.5 cm 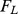 at recapture. Otoliths were also collected from 38 yellowfin captured during the WSTTP measuring between 19 to 46.6 cm 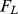 (Table S1, Fig. S1). Otoliths were extracted, rinsed in water to remove tissue, and stored dry.

Yellowfin have fragile, thin and elliptical otoliths that require particular care during their preparation and subsequent interpretation of microstructural features [48]. All the otoliths collected were analyzed at the "Laboratoire de Sclérochronologie des Animaux Aquatiques" (LASAA) in Brest, France. Otoliths were prepared for age analysis using the following method [49]; [20]; [14]. They were cleaned in sodium hypochlorite and rinsed with distilled water before being embedded in resin blocks and transversally cut on both sides of the nucleus. The sections containing the nucleus were then fixed to a glass slide using thermoplastic glue and sanded to the level of the nucleus using different alumina grains (0.3 to 3 µm). The operation was performed on each side of the section until a slice of about 100 µm thickness was decalcified with EDTA (tri-sodium-ethylene-diamine-tetra-acetic acid) to increase the contrast between increments. The thin slides were examined under a microscope (1000×magnification) to count increments along the counting path on the sagitta, i.e. from the primordium, original point of growth, to the last increment deposited on the maximal growth axis.

**Figure 1:**
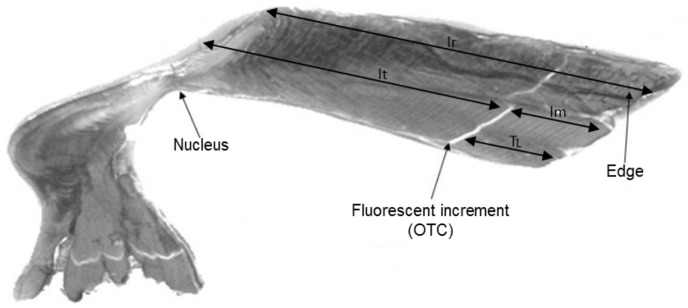
Otoliths of yellowfin tuna (external right and internal left; a) and the different sections used for reading the number of increments. OTC: Oxytetracycline; 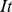: section from the nucleus to the OTC mark; 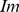: section from the OTC mark to the edge; 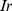: section from the nucleus to the edge; 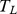: Time-at-Liberty.

Otoliths collected from OTC-tagged fishes, an increment count was made for different otolith sections: (i) between the nucleus and the OTC mark (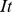), (ii) between the OTC mark and the edge (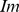) and (iii) between the nucleus and the edge (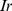) (Fig. 1). For fish that were not chemically tagged were read in full (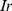). All otolith readings were performed by the same reader. Each otolith was read two to five times without prior knowledge on length or time-at-liberty of the individuals sampled so as to maintain certain independence between the multiple readings.

### Ageing error model

In this section, a hierarchical model was developed to estimate the age of each fish. The stochastic processes associated with otolith preparation and reading were modeled with the choice of an error structure and informative prior density functions based on expert judgment (Tables 1 and 2). In a first step, the hypothesis of daily increment deposition in otoliths, that has been observed in eastern Pacific yellowfin tuna [48]; [50], was tested using a subset of OTC-tagged otolith data. Information on the increment deposition process was subsequently used for estimating yellowfin age based on counts of otolith increments.

**Table 1:** Parameters and variables used in the ageing error and somatic growth models.

Variable	Definition	Equations
**Ageing error model**		
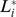	Observed fork length, i.e. length from the front to the fork in the center of the tail, for fish 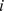 (cm)	
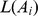	Expected fork length for fish 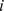 (cm)	
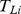	Number of days between tagging and recapture for fish 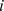 (d)	D1, D3, D5
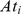	Age-at-tagging for fish 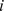 (d)	D2, D5
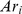	Age-at-recapture for fish 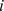 (d)	D3, D4
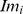	Number of increments between OTC mark and edge for otolith of fish 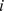	S1, D1
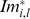	Number of increments counted between OTC mark and edge for reading 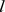 of otolith for fish 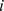	S1
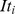	Number of increments between nucleus and OTC mark for otolith of fish 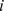	S2, D2
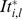	Number of increments counted between nucleus and OTC mark for reading 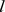 of otolith for fish 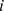	S2
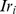	Total number of increments for otolith of fish 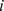	S3, D4
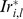	Total number of increments counted for reading 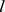 of otolith for fish 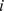	S3
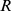	Ratio between number of increments after OTC mark and time-at-liberty	D1, D2, D4
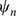	Bias at the nucleus	D1, D4
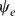	Bias at otolith edge	D2, D4
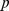	Relative percentage of misread otolith increments	S1, S2, S3
		
**Somatic growth model**		
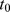	Theoretical age at fork length 0 (y)	D6, D7
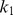	Juvenile growth rate coefficient (y 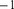 )	D6, D7
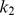	Adult growth rate coefficient (y 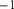 )	D6, D7
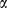	Inflection point between the 2 stanzas (y)	D6, D7
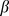	Transition rate between 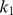 and 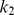	D6, D7
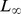	Asymptotic fork length (cm)	D7
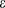	Length measurement error (cm)	S4, S5

**Table 2:** Deterministic and stochastic processes used in the ageing error and growth models. All variables are defined in table*#146;1.

Process functions	
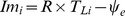	(D1)
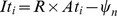	(D2)
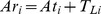	(D3)
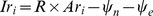	(D4)
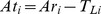	(D5)
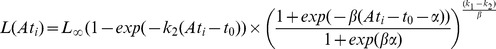	(D6)
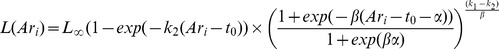	(D7)
**Observation functions**	
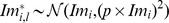	(S1)
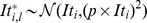	(S2)
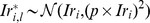	(S3)
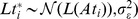	(S4)
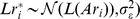	(S5)
**Prior probability distributions**	
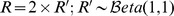	(P1)
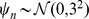	(P2)
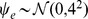	(P3)
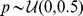	(P4)
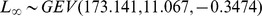	(P5)
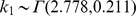	(P6)
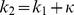 with 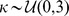	(P7)
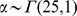	(P8)
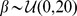	(P9)
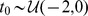	(P10)
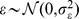 ; 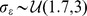	(P11)

#### Modeling observation errors

We assumed that the discrepancies between repeated readings of the same otolith mainly resulted from errors in interpreting missing increments and, to a lesser extent, from errors in counting, i.e. increment omission or multiple counts. The counting errors were considered to be equiprobable. Each increment has the same independent probability of misinterpretation, so errors tend to increase with age. In addition, the identification and interpretation of increments become increasingly difficult with increasing distance from the nucleus. Therefore, the relative reading error was considered to be dependent on true fish age and a multiplicative error was used. The relative percentage of misread increments, 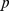, was assumed to be a constant factor uniformly distributed between 0 and 0.5 (Table 2, Eq. P4). For each reading of the same otolith, the reader was assumed to have the same probability of underestimating or overestimating the number of increments. The number of increments counted for reading 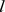 of otolith 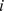 (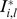) was assumed to be distributed around the expected number of increments according to a Poisson process. Here, a normal distribution was chosen to offer more flexibility in modeling uncertainty in readings and because it closely approximates the Poisson distribution for large values of the Poisson parameter according to the central limit theorem (Table 2, Eq. S1–S3).

Identifying the first growth increment is an important step in defining the starting point of ageing and accurately estimating fish age. The otolith nucleus is an opaque spot formed during embryonic development. The first increment is formed at hatching and appears as a discontinuity surrounding the nucleus of the otolith [22]. During preparation, excessive sanding of the otolith can result in the “disappearance” of the nucleus as well as the removal of some increments. Technical experts considered that up to 15 of the first otolith increments may be lost during preparation. The lost increments are then estimated with a bias of 2–3 increments (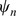, Table 2, Eq. P2).

Similar difficulties can be associated with distinguishing the marginal increment at the edge of the otolith. In this region, increments are often more difficult to read because they are narrower and can appear laterally compressed [18] In addition, the otolith must be cut perpendicularly to the daily growth axis passing by the nucleus, otherwise some increments can disappear. Here, technical experts considered that up to 20 increments may be lost at the otolith edge and an estimation bias of 3–4 increments can occur (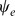, Table 2, Eq. P3).

#### Determining of the deposition periodicity

In a first step, the counts of 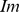 were modeled as a function of the time-at-liberty (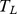), to determine the periodicity of increment deposition estimated by the reader. A subsample of 27 OTC-tagged fishes of 49.7–131 cm 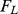 was selected to maximize reliability, i.e. individuals for which the accurate date of recapture was known and for which the coefficient of variation (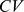) of the repeat readings of a given otolith was less than or equal to 10% [15] A Bayesian linear regression model was fitted to the data to estimate the rate of increment deposition (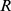) and the error at the otolith edge (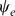) (Table 2, Eq. S1 and D1). We used a dilated beta distribution as prior for 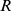 so as to provide information on the limit values without a particular trend in the distribution shape. Based on expert knowledge, an informative prior was considered for the marginal error 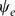 (Table 2, Eq. P3).

#### Estimating age from multiple readings

In a second step, the uncertainty associated with multiple otolith readings was modeled to estimate the true number of increments for each fish otolith. This number was then converted to age by taking into account 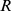. When the date of recapture was known with precision (CV
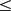
5%), the age-at-tagging (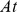) was derived from 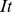 and the age-at-recapture (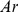) was deduced from 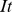 and the time-at-liberty so as to decrease the reading-associated uncertainty (Table 2, Eq. D2 and D3). When the number of increments at tagging was unknown, the age-at-recapture was derived from 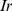 and the age-at-tagging was derived by subtracting the time-at-liberty to this number of increments (Table 2 Eq. D4 and D5). For yellowfin collected through the RTTP with low accuracy in time-at-liberty (CV
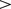
5%) and for those from WSTTP, only the age-at-recapture was estimated from 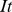. To account for uncertainty associated with the recapture dates (see Section), the time-at-liberty was considered to be a random variable with a uniform distribution between its minimal and maximal value. The posterior distributions of 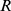 and 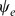, that were estimated in the previous step, were used to estimate fish age. An informative prior was considered for the nucleus bias 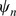. In the absence of information in the data, these distributions were not updated.

### Evaluating model performance through simulations

Different simulations were performed to test and validate the ageing error model (Fig. S2). The first simulation aimed to assess the accuracy of the model in estimating 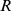. For this simulation, two alternative runs with and without individual variability in increment formation were considered, i.e. 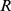 fixed to 0.95 and 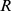 varying according to a normal distribution around 0.95. This simulation was repeated three times using datasets of 25 individuals whose time-at-liberty were generated according to a uniform distribution between 30 and 970 days. Four noisy readings of the number of 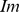 increments were then generated according to Eq. D1 and S1 (2).

A second set of simulations was performed using 2 to 5 readings of the same otolith to evaluate the model's ability to accurately estimate fish age and its relevance relative to the traditional method which is based on averaging individual-specific increment counts. An intermediate method was also considered that based on averaging individual increment counts, but also account for potential bias in the increment deposition. For these simulations, 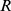 was fixed to 0.95. The approach consisted of simulating realistic ages-at-recapture from which increment counts were derived with respect to 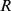, 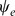, and 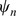 (2 Eq. D4). Noise was then added to the increment counts by randomly generating repeated readings (Eq. S3). Five age classes were considered, from 6 months to 5 years. Three simulations were performed using datasets composed of 500 individuals, i.e. 100 individuals for each age class whose ages were generated according to a uniform distribution between the minimum and the maximum values of the class. The accuracy of age estimates was assessed with the relative root mean square error (RMSE), a normalized indicator that measures the discrepancy between simulated and estimated ages. For each individual, the RMSE was calculated as follows:
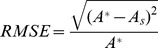
where 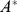 is the estimated age and 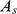 is the simulated age.

### Coupling the ageing error and the growth models

In this section, we implemented a hierarchical model that allows for growth variations according to an individual's specific stochastic process. For IO yellowfin, modal progression analysis [30]; [32]; [33] and the preliminary analysis of the RTTP data [51] indicated a succession of phases of growth deceleration and acceleration, supporting the use of a two-stanza growth model. Accordingly, we used the Von Bertalanffy logK model (VB-logK) developed for southern bluefin tuna (*Thunnus maccoyii*), which allows for a smooth transition between two different growth rate coefficients (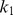 and 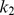) by modeling changes in growth using a logistic function [52]; [53]. The fork length of fish 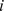 at the opportunity of capture 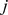, 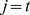 at tagging and 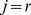 at recapture, was then modeled as:
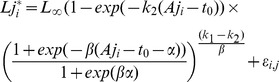
where errors in length measurements, 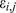, were assumed to be independent and normally distributed around zero with a common variance 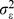.

The somatic VB-logK growth model was coupled to the ageing error model and fitted to the data using Bayesian inference (Table 2, Eq. D6 and D7). Consider the vector of growth parameters, 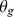 = {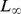, 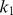, 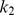, 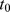, 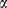, 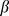, 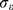}, the vector of ageing parameters 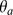 = {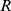, 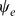, 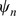}, and the relative percentage of misread otolith increments, 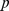. 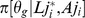, 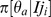, 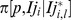 denote the posterior distributions of these parameters and 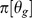, 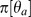, 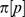 denote their prior distributions. Here, 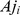 and 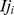 are not directly observable latent variables. The full model corresponds to the joint distribution of parameters and latent variables. As 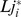 and 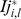 are independent, this joint posterior distribution can be written as:
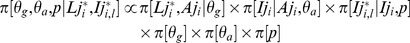
(L1)


where 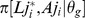 represents the conditional Gaussian likelihood of the observed lengths. Thus, the length values were predicted from the marginal posterior distribution:
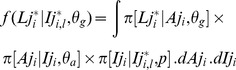
(L2)


The growth rate coefficients 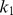 and 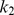 are partly model-specific, thus weakly informative priors were assigned to them. 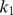 was assumed to vary according to a gamma prior distribution with a mean and coefficient of variation determined from the literature. 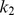 was set equal to 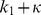 with 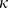 following a uniform distribution (Table 2 Eq. P6 and P7). The transition rate between 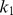 and 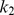, 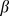, which is specific to the VB-logK model, and the theoretical age of zero length, 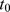, which depends on the data, were assigned uniform distributions (Eq.P9 and P10). The parameter 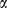 is the mean age relative to 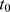 at which the change in growth occurs. This was assigned a weakly informative prior gamma distribution with a mean defined from the literature (Eq. P8; [36]; [37]; [32]; [33]). The standard deviation of size measurement errors, 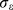, was determined from the differences in the fork length of RTTP-IO fish released and recaptured several times with time-at-liberty less than or equal to seven days. These individuals were not included in subsequent analyses and therefore constitute an independent data set (Eq. P11).

The asymptotic length, 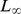, is a particularly important parameter because it determines the shape of the second part of the growth curve. As the dataset included limited information on the asymptotic part of the growth curve, auxiliary information was provided for this parameter consistently with the available knowledge on the biology of the species. An informative prior distribution was defined for 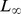 using a generalized extreme value distribution (GEV). This allows for the extrapolation of the behavior of distribution tails from the greatest values of a sample and thus estimates the probability of the occurrence of extreme events [54]. The choice of this distribution is motivated by the fact that yellowfin grow throughout their life, such that the largest observed sizes should correspond to the oldest fish. The distribution was fit based on size measurement data from fresh fish that were collected during 1952–2011 from the European and Seychelles purse seine fisheries, Maldivian pole and line vessels, and Taiwanese and Japanese longliners. The observed maximum fork length from each measurement platform, i.e. either on vessels board or in cannery, fishery, and year was considered to represent 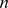 independent random variables (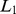,...,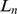) with common continuous distribution function 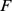. Asymptotic length 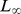 was then estimated from the approximation of the upper tail of 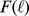 by using the 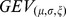 distribution:
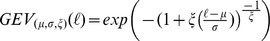
where 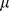 is a location parameter, 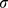 is the scale parameter (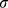 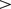
0) and 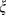 is a tail index (shape parameter). These parameters were estimated using the maximum likelihood method [55].

The estimates of ages and growth parameters were evaluated based on 350,000 samples, thinned to one draw every 1000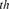 sample, from Markov Chain Monte Carlo (MCMC) simulations of the joint posterior distribution. A burn-in period of 5,000 iterations was rejected. Three MCMCs were produced using a Gibbs sampler as implemented in OpenBugs version 3.2.1 [56]. The convergence of the MCMC to a stationary posterior distribution was visually evaluated from the Gelman-Rubin diagnostic, which was based on the ratio of inter-chain variance on intra-chain variance, i.e. it must be close to one in order to converge [57].

### Testing the influence of the ageing technique on growth modeling

Simulations were performed to evaluate the contribution of the error model on growth parameter estimates and resulting age classification of commercial catches. To illustrate, a simple tagging dataset was simulated and the relationship specified from posterior marginal mode that was obtained with the growth model coupled with the ageing error model (section and Table 3) was used as reference growth curve. In this approach, four fork lengths for each 2 cm class, from 20 to 146 cm, were simulated to obtain a data set of 252 fishes. As the VB-logK curve is irreversible, corresponding ages were deduced by minimizing the difference between the simulated and expected length (E1). These ages were converted into numbers of increments from which noisy repeated readings were generated as per the approach previously developed (section and Fig. S2). The growth parameters were then re-estimated using the coupled VB-logK model and a classical VB-logK model, which estimates age using the traditional method. As the parameter 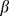 is difficult to estimate from a tagging dataset, its value was fixed using the value obtained by fitting the otolith data to the coupled growth model.
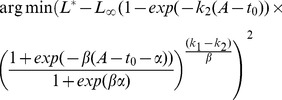
(E1)


**Table 3:** Attributes of marginal posterior distributions from VB log K growth model coupled with ageing error model fit to yellowfin otolith data.

Parameters	Mode	Mean	Std.dev*^a^*	Posterior quantiles 2.5% 97.5%	
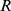 (days)	0.939	0.94	0.029	0.8834	0.9983
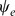	−0.54	−0.549	2.469	−5.404	4.263
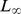 (cm)	146.243	151.809	15.963	126.6	188.4
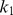	0.246	0.249	0.04	0.1745	0.3283
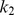	0.664	0.878	0.434	0.371	2.114
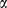 (years)	2.61	2.625	0.162	2.353	2.951
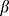	12.583	11.663	4.807	3.718	19.58
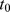 (years)	−0.43	−0.446	0.088	−0.642	−0.3022
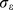 (cm)	8.809	8.876	0.557	7.864	10.05

a Standard deviation

The mean squared error (MSE) was used to compare the marginal posterior distributions of each parameter with its actual value and evaluate the accuracy of models fits. Accuracy is related to the similarity between the marginal modes and the true values, while precision refers to the uncertainty around the modal value, i.e. the discrepancies between the samples generated by the MCMC simulation. The MSE is a measure of the average of the square of errors and was calculated as follows:
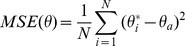
where 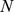 is the size of the MCMC sample, 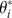 is the parameter estimate at iteration 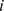 and 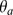 is the actual value of the parameter.

In addition, the mean relative error (MRE) was used to give information on the bias in parameter estimates:
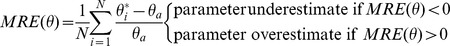



Finally, an age-length key was derived from both the coupled model and the classical model and was used to convert the catch-at-size into catch-at-age. The two conversions were then compared. For this example, we used 2010 IOTC catch data from French purse seiners in the Western Indian Ocean.

## Results

### Simulation evaluation

The fit of the ageing model to the simulated data showed good agreement between the estimated and simulated age, as well as a strong ability to estimate the increment deposition rate 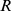. From the simulations conducted in the first model step, the marginal posterior distribution of 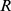, derived from MCMC outputs, followed a normal distribution with a mode and variance very close to the simulated distribution. The fixed simulated values were well within the 95% credibility interval of the posterior distributions (Fig. S3). The model showed difficulties in estimating the edge bias 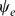 by systematically underestimating its variability.

Simulations that focus on the number of readings showed that the ageing error model provided accurate age estimates, with accuracy increasing with the number of readings (Fig. S4). The RMSE values were significantly lower when there were more readings of the same otolith (Wilcoxon: p-value
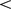
0.05; Table S2). From five to three readings, the loss of accuracy was generally low, i.e. an average loss of 1%. However, the loss of accuracy became more important between three to two readings, suggesting that three readings was a good compromise between accuracy of estimates and reading cost.

Regardless of the number of readings, the ageing error model estimated age more accurately than the traditional method. The RMSE values obtained with the ageing error model were significantly lower than those obtained with the traditional method (Wilcoxon: p-value
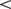
0.01; Fig. S5 and Table S3). The ageing error model and the intermediate ageing method provided age estimates as accurate. The RMSE values obtained with the ageing error model and the intermediate method did not differ significantly (Table S3), with the exception of second simulation dataset where the RMSE values significantly lower with the ageing error model (Wilcoxon: p-value
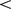
0.01).

### Testing the hypothesis of daily increment deposition

The ratio 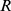 and the mean edge bias 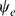 were precisely estimated in the regression model (Fig. S3). 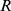 was normally distributed around 0.94 with little variability (Table 3). The value of one was not included in the Bayesian 95% credibility interval which resulted in a significant failure of the hypothesis of daily increment deposition. The reader tended to underestimate the fish ages. The edge bias was close to zero, with a lower variability than expected from the prior distribution, resulting in error values ranging from 1.6% for a six month-old fish to 0.16% for a five year-old fish (Table 3).

### Yellowfin tuna growth

**Figure 2:**
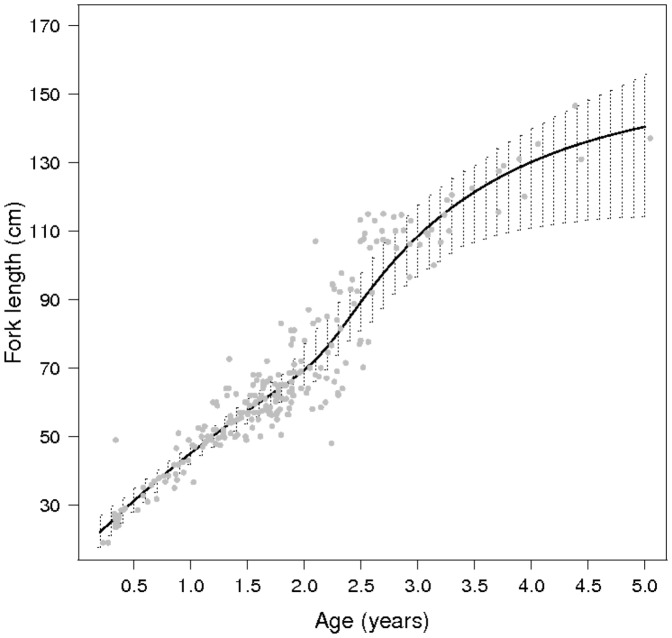
Yellowfin growth curve as estimated from the VB log K model coupled with the ageing error model. The solid line correspond to the mean growth curve, the dashe to the uncertainty around the mean curve and the points to observation data.

The model supported a two-stanza growth for the IO yellowfin with two distinct phases over the fish lifespan of the fish (Figure 2). The first stanza was characterized by relatively slow growth, which decreased gradually to a minimum of 1.75 cm.month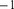 for fish up to 1.85 years of age (63 cm 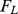). In the second stanza, growth accelerated up to a maximum of 3.54 cm.month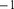 for fish up to 2.38 years of age (79 cm 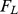) and then progressively decreased with size, becoming very slow when the size approached the asymptotic length.

The fit of the coupled growth model revealed strong correlations among some parameters. A positive correlation was found between 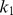 and 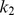 (0.8) and a negative correlation was found between these growth rate coefficients and the asymptotic length (−0.93 and −0.8 with 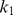 and 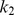 respectively; Table S4). These correlations resulted in some model instability and difficulty fitting the posterior distributions of the parameters, particularly in the absence of information from the prior distribution (Fig. S6). The expected asymptotic length was estimated at 151.8 cm 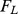, which was close to the maximum fork length of the data, 146.5 cm 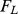, but comparatively low to the mean asymptotic fork length estimated at about 173 cm from the catch of the purse seiners and longliners and to the maximum lengths of 200 cm that has been observed for yellowfin in the Indian Ocean. In contrast, the data provided more information for the first part of the growth curve (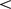
3 years), where the model fit was good. The uncertainty associated with the mean growth curve that was derived from marginal posterior modes increased as the fish aged.

### Sensitivity of growth parameters to biased age estimates

Except for 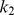, the MRE values derived from the simulations indicated that estimates were more accurate for the coupled model than for the classical model (Table 4). For both models, 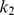 was estimated with a negative bias of an average value of 19% and 10% for the coupled and classical models respectively. 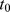 was estimated with a positive bias for the classical model. For the other parameters, the mean biases were less than 10%. The MSE values were high for the asymptotic length 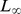. The influence of the GEV prior distribution was greater for the asymptotic part of the curve than for the fit to the real data as this part had very few data points. This resulted in the asymptotic lengths being overestimated. For the other parameters, the MSE values were low (Table 4). The MSE values for the estimates of 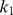, 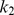 and 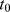 were higher for the coupled model than for the classical model indicating a greater variability in the estimates of the coupled model. These results suggested that the coupled model provided more accurate, but less precise, estimates than the classical model because it accounted for uncertainties associated with age estimates while the ages were fixed in the classical model.

**Table 4:** Features of both growth models fit to simulated data.

Parameters	Coupled growth model							Classical growth model						
	Mode	Mean	Std.dev *^a^*	Posterior quantiles 2.5% 97.5%		MSE	MRE	Mode	Mean	Std.dev	Posterior quantiles 2.5% 97.5%		MSE	MRE
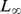 (cm)	148.288	149.433	4.940	141.3	160.9	35.517	0.022	149.15	150.465	6.952	146	185.3	64.900	0.029
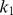	0.252	0.247	0.029	0.2042	0.2831	0.0008	0.005	0.264	0.262	0.009	0.2374	0.2777	0.0003	0.065
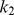	0.535	0.539	0.084	0.3815	0.7136	0.023	−0.190	0.604	0.596	0.072	0.2463	0.6827	0.009	−0.101
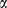 (years)	2.482	2.481	0.153	2.161	2.774	0.040	−0.051	2.438	2.538	0.579	2.338	5.228	0.308	−0.029
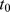 (years)	−0.398	−0.438	0.223	−0.5863	−0.3146	0.0461	0.015	−0.376	−0.374	0.028	−0.4183	−0.2746	0.004	−0.127

* Standard deviation.

**Figure 3:**
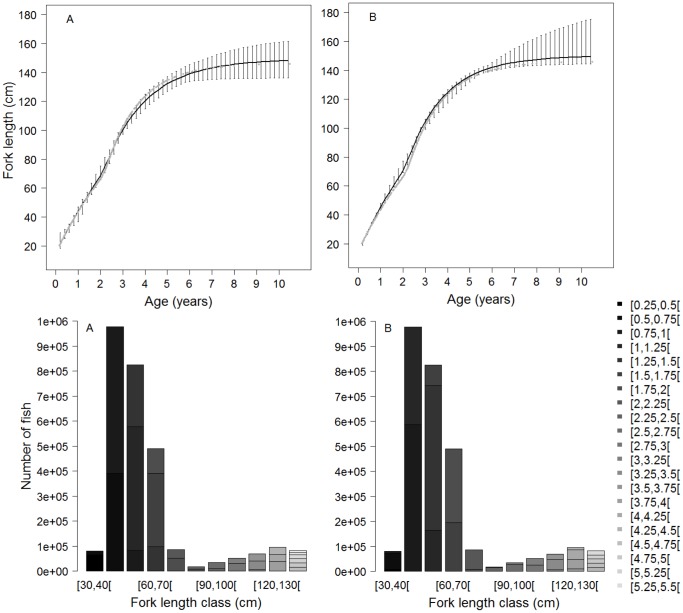
Growth curves obtained from the fit to simulated data with the coupled growth model (A) and the classical growth model (B; up) and their use to convert the size frequencies from fishing catches into age frequencies (down). Gray levels correspond to the age classes (quarter).

The growth curve obtained from the coupled model fit coincided with the simulated data in the first stanza but tended to underestimate the growth between 3.5 and 6.5 years. The growth curve of the classical model consistently overestimated the simulated growth (Figure 3). Such deviations in growth patterns led to a significant divergence in the age-length key that was derived from both models. This divergence resulted in potentially major differences when converting catch-at-size into catch-at-age. As illustrated by the 2010 yellowfin catch data, the proportions-at-age by 10 cm class lengths were significantly different according to the age-length key, particularly for lengths of less than 100 cm (Figure 3).

## Discussion

Otoliths have been widely used to study fish species that consistently deposit growth increments over time [13]; [58]; [59]; [11]. However, estimating fish age by counting of otolith increments can result in large biases and uncertainties due to the combination of processing and interpretation errors [18]. Both error types affect the estimates of growth, mortality and other demographic rates required for population dynamics models, e.g growth, mortality. To our knowledge, only a few studies have previously attempted to quantify errors in age estimates. In this study, we have developed an ageing error model that explicitly accounts for these potential errors, thus improving age estimation and propagating the uncertainty that arises from otolith reading into the estimation of fish growth. We applied our model to Indian Ocean yellowfin tuna, drawing on a dataset of repeat otolith readings that were collected during large mark-recapture experiments. To illustrate the interest of our method and assess the potential consequences for input data used in fish stock assessment models, we completed a series of simulations. Through Bayesian analysis, the uncertainty associated with age estimates derived from hard structures can be combined with supplementary information, e.g., observations of the maximum size, or growth estimates for the same species in other areas, and expert knowledge to estimate growth.

### Age estimation method

The traditional ageing method, which converts an average count of increments into age, depends on the assumption that each increment corresponds to one day [60]. Otherwise, the use of this method will lead to systematically biased age estimates. In the case of the European hake (*Merlucccius merluccius*), it has recently been demonstrated that the assumption of a daily deposition rate is not met. As such, although the use of the traditional method for this species is internationally accepted, its use has resulted in a significant overestimation of fish age with important implications for estimates of key biological parameters [61]. This example highlights that validation of the increment deposition rate for the entire age range of interest should be major prerequisite for any ageing study. Various age validation techniques exist including the release of tagged fish of a known age, nuclear or radiochemical dating, modal progression of catch-at-size data over time, and captive rearing [25]. For yellowfin, the assumption of daily deposition was tested through a mark-recapture experiment of wild fish with oxytetracycline (OTC) in the length interval from 49.7–131 cm 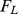. The result of this experiment suggested that the deposition rate estimated by the reader within this length interval is significantly different than the assumed single increment per day, with an increment-over-day ratio of 1∶0.939. The marginal posterior distribution revealed little variation in this ratio, suggesting that the reading method tended to systematically underestimate the actual fish ages. However, additionnal readings performed by new readers for some of the otolith data used in the present study however indicate a 1∶0.997 ratio consistent with a daily deposition rate as shown for the Pacific Ocean yellowfin [48]. As tuna otoliths are structurally complex, a high degree of interpretation is required reading when them, which can lead to a reading bias and may account for the differences in estimated deposition rates [62]; [63]. This highlights the need for inter-comparison exercises between research institutes, using reference otolith collections, so as to harmonise reading methods [23]. This first step of the model can be adapted to other species or to other age validation techniques.

It is also necessary to assess the precision of the ageing technique [25]. The main uncertainty in age estimates arises from discrepancies between repeated readings. The magnitude of this uncertainty is partly dependent on the proficiency of the reader [16]. Thus, when a calcified structure is read by various readers, our model must be adapted to take account of the probability of misinterpretation of each reader. In addition, for some otoliths, the interpretations of the first and marginal growth increments can generate further uncertainty, particularly as these rings may obliterate during a poor otolith preparation. These additional uncertainties were relfected in the model through the choice of stochastic error structures and informative prior distributions based on the expertise to compensate for the lack of information in the data. In this context, Bayesian analysis appears particularly adapted to include such information into the statistical inference procedure.

The model evaluation by simulation suggested that it was capable of estimating both the ageing biases and an accurate age regardless of the age of the fish. The mean and variance of the deposit bias were consistently well-estimated. The model performed less well in estimating the bias linked to the misinterpretation of the marginal rings. However, edge level errors are partly estimated by the overall reading error. For yellowfin, expert judgement suggests that the nucleus and edge biases could be very low, i.e. less than five increments, resulting in error ranging from 4% for a six month-old fish to 0.4% for a five year-old fish. As such, these biases would become negligible when the age in days values are converted to age in year values. Thus, the intermediate method in which the average number of increments is corrected for the actual deposit frequency provided age estimates as accurately as the ageing error model. However, the range of uncertainty is fully ignored which may have serious implications on the final outcome of the analysis [43].

### Growth of Indian Ocean yellowfin

The method developed in this study appears suitable for modeling the growth of Indian Ocean yellowfin. The fit of the growth model appeared to be adequate in the first stanza. In this first phase, the expected first growth rate, 2.11 cm.month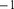 for fish from 22 to 63 cm 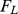, and length from which growth accelerates, 63 cm 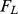, were consistent with several growth studies for the same population. Growth rates of small yellowfin have been estimated to be between 1.3 and 2.9 cm.month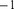 [30]; [63]; [31]; [65]; [32] with growth acceleration occurring around 62 to 66 cm 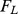 [31], 70 cm 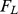 [65], 56 to 66 cm 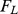 [32] and from 60 cm 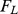 [33]. However, in the absence of older fish, the model seemed to underestimate the expected asymptotic length leading to an apparent underestimation of yellowfin growth over 2.7 years of age (95.72 cm 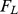). Increasing the uncertainty in the second stanza reflected greater individual variability in growth. A primary source of variability in growth rates may arise from sexual dimorphism, characterized by faster growth and a larger asymptotic length for males than females [66]; [36]. According to Wild [60], in the eastern Pacific, yellowfin females grow faster than males until 94.9 cm 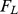, i.e. at about 2 years old, and then the trend reverses. Several authors have also shown that in IO yellowfin, males are largely dominant above 145 cm 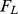 [67]; [68] which is close to the modal value of the asymptotic length estimated in this study at 146.5 cm 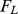.

Estimating the growth parameters with the growth model coupled with the ageing error model was more accurate, but less precise, than with the classical growth model. This latter used the traditional ageing method which underestimated the “actual” age of fish and resulted in a consistent overestimation in the growth curve. In contrast, taking into account uncertainties associated with age estimates led to a growth curve that fitted very closely to the simulated data in the first stanza. Such differences greatly influenced the age distributions of the fisheries catches. In addition, by disregarding the ageing errors, the classical model was overconfident in determining uncertainties of growth estimates. It is critical to represent the full range of uncertainty to adequately evaluate management alternatives and the expected consequences of decision-making [69]; [70]. Developed within a hierarchical Bayesian framework, our method appears particularly suited to quantifying the uncertainties resulting from otolith reading or observational errors and their propagating in the estimates of biological parameters.

However, there are some technical difficulties associated with the use of Bayesian inferences. One of the main criticisms is the need to specify prior distributions, which can have some influence on the parameter posterior distributions [71; 38]. Commonly, sensitivity analyses are performed to evaluate the effect of prior change on the posterior. As increasing data quality and quantity reduces the prior influence. Therefore, these sensitivity analyses are above all an assessment tool for appreciate the amount of information in the data [40]. In this study, extensive work was undertaken on prior specification and weakly informative distributions were assigned to the parameters for which there was no established scientific understanding. This was done to obtain estimates that were more consistent with the biology of yellowfin.

The general approach developed in this study can be applied to other species and can be adapted to any structure that produces periodic growth increments in response to various ecological issues.

## Supporting Information

Figure S1 Tagging area (gray-colored) and points of tag recovery (circles) of RTTP program and sampling area of WSTTP program (square).
(TIF)

Figure S2 **Simulation framework for testing the ageing error model.** Different sources of uncertainty are added to simulated ages to randomly generate noisy increments that were then used as inputs in the model.
(TIF)

Figure S3 **Marginal posterior distributions of the ageing error model parameters (black) compared with the simulated values (grey).** Two alternatives were considered, with (right) and without (left) individual variability in increment formation; A, B and C represents the first, second and third simulated data set respectively.
(TIFF)

Figure S4 Boxplot of the RMSE values obtained with the ageing error model for different number of otolith readings. a, b and c represents the first, second and third simulated data set respectively.
(TIFF)

Figure S5 Boxplot of RMSE values obtained with the ageing error model, the traditional method and the intermediate method for different number of otolith readings. a, b and c represents the first, second and third simulated data set respectively.
(TIFF)

Figure S6 Marginal posterior distributions of the parameters of the growth model coupled with the ageing error model (black). The grey curves correspond to the prior distributions.
(TIFF)

Table S1
**Summarize of data used in ageing error model.** A: fish for which the time-at-liberty is known, B: fish for which the time-at-liberty is unknown, 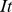: section between nucleus and OTC mark, 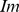: section between OTC mark and edge, 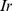: section between nucleus and edge.(DOC)

Table S2 **Comparison of RMSE values obtained with the ageing error model for different number of otolith readings using a Wilcoxon test.** 2 L, 3 L, 4 L and 5 L correspond to the number of readings of the same otolith; a, b and c represents the first, second and third simulated data set respectively.
(DOC)

Table S3 Comparison of the RMSE values obtained with the ageing error model, the traditional method and the intermediate method for different number of otolith readings using a Wilcoxon test. 2 L, 3 L, 4 L and 5 L correspond to the number of readings of the same otolith; a, b and c represents the first, second and third simulated data set respectively.
(DOC)

Table S4 Correlation-covariance matrix of growth parameters. Numerals in bold represent the covariances.
(DOC)
