# Supplementary figures and images for: Accounting for Age Uncertainty in Growth Modeling, the Case Study of Yellowfin Tuna (Thunnus albacares) of the Indian Ocean
Source: PLoS One. 2013 Apr 23;8(4):e60886. doi: 10.1371/journal.pone.0060886 (PMC3634046; doi:10.1371/journal.pone.0060886)

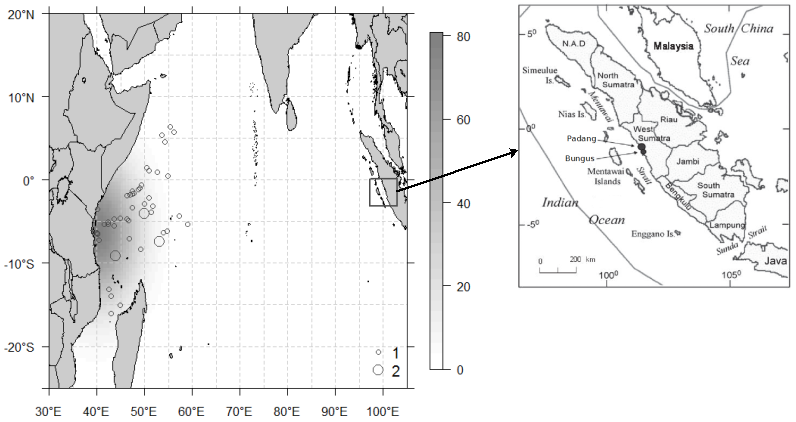

Supplement: Figure S1 — Tagging area (gray-colored) and points of tag recovery (circles) of RTTP program and sampling area of WSTTP program (square). (TIF) [file pone.0060886.s001.tif]

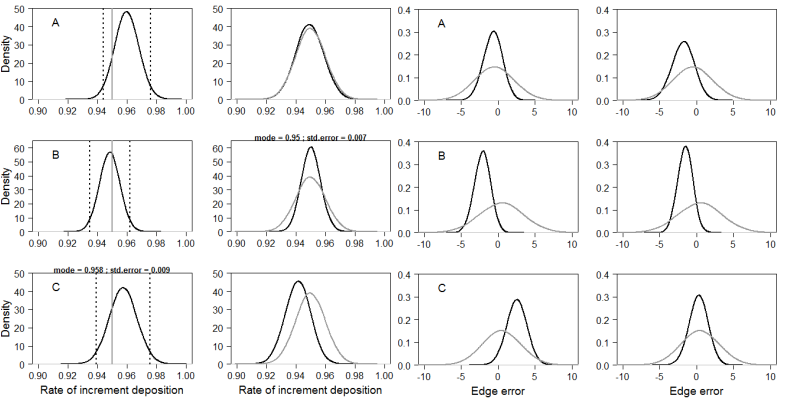

Supplement: Figure S3 — Marginal posterior distributions of the ageing error model parameters (black) compared with the simulated values (grey). Two alternatives were considered, with (right) and without (left) individual variability in increment formation; A, B and C represents the first, second and third simulated data set respectively. (TIFF) [file pone.0060886.s003.tiff]

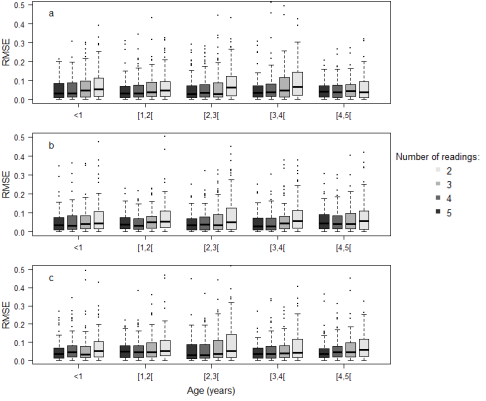

Supplement: Figure S4 — Boxplot of the RMSE values obtained with the ageing error model for different number of otolith readings. a, b and c represents the first, second and third simulated data set respectively. (TIFF) [file pone.0060886.s004.tiff]

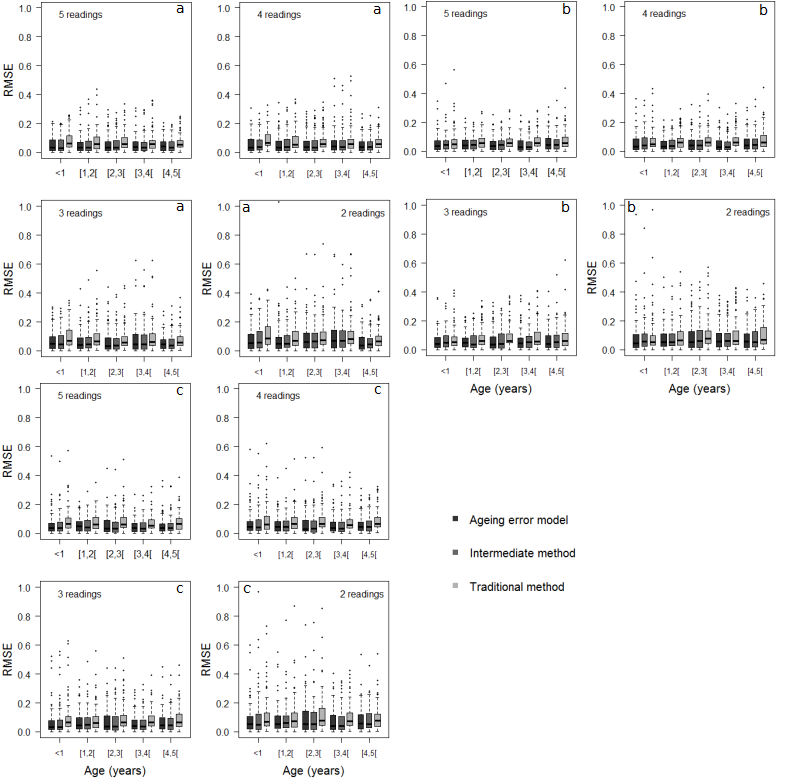

Supplement: Figure S5 — Boxplot of RMSE values obtained with the ageing error model, the traditional method and the intermediate method for different number of otolith readings. a, b and c represents the first, second and third simulated data set respectively. (TIFF) [file pone.0060886.s005.tiff]
