# Supplementary material for: Accounting for Age Uncertainty in Growth Modeling, the Case Study of Yellowfin Tuna (Thunnus albacares) of the Indian Ocean
Source: PLoS One. 2013 Apr 23;8(4):e60886. doi: 10.1371/journal.pone.0060886 (PMC3634046; doi:10.1371/journal.pone.0060886)
Supplement: Table S1 — Summarize of data used in ageing error model. A: fish for which the time-at-liberty is known, B: fish for which the time-at-liberty is unknown, : section between nucleus and OTC mark, : section between OTC mark and edge, : section between nucleus and edge. (DOC) [file pone.0060886.s007.doc]

**Table S1. Summarize of data used in ageing error model.** A: fish for which the time-at-liberty is known, B: fish for which the time-at-liberty is unknown, *It*: section between nucleus and OTC mark, *Im*: section between OTC mark and edge, *Ir*: section between nucleus and edge

|  | RTTP | | | | WSTTP |
| --- | --- | --- | --- | --- | --- |
|  | A | | | B | B |
| Otolith section | *It* | *Ir* | *Im* | *It* | *It* |
| Number of fish | 80 | 14 | 27 | 30 | 38 |
| Fork length at tagging (cm) | 43 to 85 | 50 to 72 |  |  |  |
| Fork length at recapture/capture (cm) | 49.7 to 135.4 | 59.5 to 114.1 | 49.7 to 131 | 47.9 to 146.5 | 19 to 46.6 |
| Time-at-liberty (days) | 43 to 969 | 33 to 414 | 43 to 969 |  |  |
