# Supplementary material for: Accounting for Age Uncertainty in Growth Modeling, the Case Study of Yellowfin Tuna (Thunnus albacares) of the Indian Ocean
Source: PLoS One. 2013 Apr 23;8(4):e60886. doi: 10.1371/journal.pone.0060886 (PMC3634046; doi:10.1371/journal.pone.0060886)
Supplement: Table S2 — Comparison of RMSE values obtained with the ageing error model for different number of otolith readings using a Wilcoxon test. 2 L, 3 L, 4 L and 5 L correspond to the number of readings of the same otolith; a, b and c represents the first, second and third simulated data set respectively. (DOC) [file pone.0060886.s008.doc]

**Table S2.1. Comparison of RMSE values obtained with the ageing error model for different number of otolith readings using a Wilcoxon test.** 2L, 3L, 4L and 5L correspond to the number of readings of the same otolith; a, b and c represents the first, second and third simulated data set respectively

|  |  | 4L | 3L | 2L |
| --- | --- | --- | --- | --- |
| a | 5L | V = 54527  p-value = 0.01224 |  |  |
|  | 4L |  | V = 48750  p-value = 1.767e-05 |  |
|  | 3L |  |  | V = 45892  p-value = 2.260e-07 |
| b | 5L | V = 54789  p-value = 0.01535 |  |  |
|  | 4L |  | V = 69539  p-value = 0.0324 |  |
|  | 3L |  |  | V = 45324  p-value = 8.683e-08 |
| c | 5L | V = 46959  p-value = 1.257e-06 |  |  |
|  | 4L |  | V = 69539  p-value = 0.0324 |  |
|  | 3L |  |  | V = 45324  p-value = 8.683e-08 |
