# Supplementary material for: Accounting for Age Uncertainty in Growth Modeling, the Case Study of Yellowfin Tuna (Thunnus albacares) of the Indian Ocean
Source: PLoS One. 2013 Apr 23;8(4):e60886. doi: 10.1371/journal.pone.0060886 (PMC3634046; doi:10.1371/journal.pone.0060886)
Supplement: Table S3 — Comparison of the RMSE values obtained with the ageing error model, the traditional method and the intermediate method for different number of otolith readings using a Wilcoxon test. 2 L, 3 L, 4 L and 5 L correspond to the number of readings of the same otolith; a, b and c represents the first, second and third simulated data set respectively. (DOC) [file pone.0060886.s009.doc]

**Table S2.2. Comparison of the RMSE values obtained with the ageing error model, the traditional method and the intermediate method for different number of otolith readings using a Wilcoxon test.** 2L, 3L, 4L and 5L correspond to the number of readings of the same otolith; a, b and c represents the first, second and third simulated data set respectively

|  | |  | Traditional method | Intermediate method |
| --- | --- | --- | --- | --- |
| a | Ageing error model | 2L | V = 28741, p-value < 2.2e-16 | V = 55196, p-value = 0.02155 |
|  |  | 3L | V = 28165, p-value < 2.2e-16 | V = 57314, p-value = 0.1004 |
|  |  | 4L | V = 28080, p-value < 2.2e-16 | V = 59300, p-value = 0.3037 |
|  |  | 5L | V = 28796, p-value < 2.2e-16 | V = 59429, p-value = 0.3229 |
|  | Intermediate method | 2L | V = 17140, p-value < 2.2e-16 |  |
|  |  | 3L | V = 16009, p-value < 2.2e-16 |  |
|  |  | 4L | V = 16139, p-value < 2.2e-16 |  |
|  |  | 5L | V = 18954, p-value < 2.2e-16 |  |
| b | Ageing error model | 2L | V = 37313, p-value = 4.85e-15 | V = 52640, p-value = 0.002009 |
|  |  | 3L | V = 35248, p-value < 2.2e-16 | V = 49274, p-value = 3.623e-05 |
|  |  | 4L | V = 32274, p-value < 2.2e-16 | V = 53918, p-value = 0.007069 |
|  |  | 5L | V = 35057, p-value < 2.2e-16 | V = 55195, p-value = 0.02153 |
|  | Intermediate method | 2L | V = 36636, p-value = 8.97e-16 |  |
|  |  | 3L | V = 35714, p-value < 2.2e-16 |  |
|  |  | 4L | V = 29036, p-value < 2.2e-16 |  |
|  |  | 5L | V = 32230, p-value < 2.2e-16 |  |
| c | Ageing error model | 2L | V = 26437, p-value < 2.2e-16 | V = 60299, p-value = 0.4719 |
|  |  | 3L | V = 26608, p-value < 2.2e-16 | V = 62971, p-value = 0.9149 |
|  |  | 4L | V = 22487, p-value < 2.2e-16 | V = 67331, p-value = 0.1455 |
|  |  | 5L | V = 23658, p-value < 2.2e-16 | V = 68155, p-value = 0.08714 |
|  | Intermediate method | 2L | V = 24027, p-value < 2.2e-16 |  |
|  |  | 3L | V = 19659, p-value < 2.2e-16 |  |
|  |  | 4L | V = 14297, p-value < 2.2e-16 |  |
|  |  | 5L | V = 15527, p-value < 2.2e-16 |  |
