# Supplementary material for: Accounting for Age Uncertainty in Growth Modeling, the Case Study of Yellowfin Tuna (Thunnus albacares) of the Indian Ocean
Source: PLoS One. 2013 Apr 23;8(4):e60886. doi: 10.1371/journal.pone.0060886 (PMC3634046; doi:10.1371/journal.pone.0060886)
Supplement: Table S4 — Correlation-covariance matrix of growth parameters. Numerals in bold represent the covariances. (DOC) [file pone.0060886.s010.doc]

**Table S3. Correlation-covariance matrix of growth parameters.** Numerals in bold represent the covariances

|  | 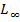 | 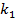 | 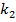 | 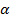 | 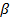 | 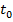 |
| --- | --- | --- | --- | --- | --- | --- |
| 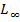 | **254.808** | -0.93 | -0.8 | -0.29 | -0.027 | -0.43 |
| 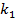 | **-0.593** | **0.002** | 0.8 | 0.21 | 0.093 | 0.67 |
| 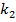 | **-5.527** | **0.014** | **0.189** | 0.48 | -0.12 | 0.34 |
| 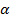 | **-7.568** | **0.001** | **0.034** | **0.026** | -0.3 | -0.074 |
| 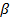 | **-2.044** | **0.018** | **-0.259** | **-0.236** | **23.11** | 0.17 |
| 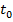 | **-0.601** | **0.002** | **0.013** | **-0.001** | **0.074** | **0.007** |
